# The evolution of moral rules in a model of indirect reciprocity with private assessment

**DOI:** 10.1038/s41598-021-02677-2

**Published:** 2021-12-08

**Authors:** Cedric Perret, Marcus Krellner, The Anh Han

**Affiliations:** Teesside University, Southfield Rd, Middlesbrough, TS1 3BX UK

**Keywords:** Evolutionary theory, Social evolution

## Abstract

Moral rules allow humans to cooperate by indirect reciprocity. Yet, it is not clear which moral rules best implement indirect reciprocity and are favoured by natural selection. Previous studies either considered only public assessment, where individuals are deemed good or bad by all others, or compared a subset of possible strategies. Here we fill this gap by identifying which rules are evolutionary stable strategies (ESS) among all possible moral rules while considering private assessment. We develop an analytical model describing the frequency of long-term cooperation, determining when a strategy can be invaded by another. We show that there are numerous ESSs in absence of errors, which however cease to exist when errors are present. We identify the underlying properties of cooperative ESSs. Overall, this paper provides a first exhaustive evolutionary invasion analysis of moral rules considering private assessment. Moreover, this model is extendable to incorporate higher-order rules and other processes.

## Introduction

Morality states which action can be considered good, which action is deemed to be rewarded and which action should be punished. Moral rules are pervasive in human societies. They can be observed in a range of examples from the behaviours of 8 month-old infants^1^ to the moral norms of societies^2^. The pervasiveness of these rules could be explained by their capacity to create cooperation by indirect reciprocity^3^. Indirect reciprocity describes a form of reciprocity where the action of an individual is reciprocated by a third party in future interactions. In its simplest form, cooperators get rewarded by receiving future cooperation, and defectors get punished by future defection^4^. Indirect reciprocity can be beneficial as it is one of the few mechanisms that can create cooperation^3,5,6^ even when the interactions are not repeated or between related individuals.

Indirect reciprocity explains well the emergence of moral rules but it is not clear which moral rules best implement indirect reciprocity, and thus, which moral rules should be observed in the real world. The number of possible rules can quickly become staggering. When individuals judge the action of another, they can take into account not only the action observed, but also the reputation of the individuals involved in the interaction. For instance, helping someone is generally seen as a positive action, but helping a criminal can be deemed bad. Do individuals use only a few rules among all the possible rules, or do a wide variety of rules coexist? Are there features common to all these rules and if yes, what are they? For instance, ones could expect that successful rules are simple ones as observed in direct reciprocity^7,8^, while others argued that rules could be so complex that it drove the evolution of larger brains^9^.

Tackling this problem, previous works have compared the evolutionary success of a large number of rules. Their results show that only few strategies stand out in term of evolutionary success and the frequency of cooperation they enforce^4,10,11^. These previous works have been a major contribution but its conclusions are limited. First, they did not consider the evolution of different assessment rules, i.e. how an individual is judged. Assessment rules were fixed in a group by moral norms, and all individuals within a group judge someone else actions in the same way. Although individuals within a group can share moral rules because they conform to common norms, evidence suggests that moral rules are also strongly determined by individual characteristics and thus, can differ between individuals. For instance, infants^12,13^ and toddlers, which were almost not exposed to social norms, already exhibit signs of indirect reciprocity^14,15^. Second, these previous models consider that the opinions and assessments are public. This means that individuals are considered either exclusively good or exclusively bad by all others at a given time. Yet, in the real world, individuals can disagree in their judgements because they have different moral rules or because they get contradictory information. For instance, hunter-gatherers exhibit reciprocity and moral^16^ , but often disagree on who exhibit these moral values^17^.

The limits of the assumption of public assessment are well acknowledged but models considering private assessment have been limited by analytical complexity. Indeed, disagreement between individual opinions can itself result in future interactions being judged differently by the actor and an observer, fuelling more disagreement. As a result, previous work that considered private assessment or individual assessment rules limited their analysis to a small set of strategies, usually the ones that have been shown successful in models with public assessment^18–21^. Recently,^22^developed a model to explore the success of a large number of assessment rules against strategies that always cooperate or defect^23^. Yet, an exhaustive study which confronts all possible rules with each other is still missing.

In this paper, we aim to fill this gap by identifying the evolutionary stable strategies among all possible moral rules. The contributions of this paper are two-fold. First, we provide the first exhaustive evolutionary invasion analysis of moral rules considering private assessment. We show that few moral rules stand out and we identify the common features of these rules. Second, we provide a model which describes the dynamics of opinions and provide the frequency of cooperation of an individual given its strategy with private assessment. This model can be extended to incorporate higher order rules and other processes, e.g. communication^24^.

## Model description

The model describes a well-mixed and infinitely large population of individuals that play a one-shot dyadic donation game. In this game, a randomly chosen individual called ‘donor’ decides whether to cooperate with another randomly chosen individual called ‘recipient’. If the donor cooperates, it pays a cost *c* to provide a benefit *b* to the receiver, while if it defects nothing happens. This game is a social dilemma as we consider $b>c$, because all would benefit if all individuals donated, but individuals are not willing to pay the cost.

Individuals hold private opinions on each other individual except themselves. Opinions are either 1 or 0, i.e. good or bad. Individuals use these opinions to apply their strategies. A strategy consists of a set of action rules, *A*, and two sets of assessment rules, *C* and *D*,

1 $$\begin{aligned} A = \begin{pmatrix} a_{1} \\ a_{0} \\ \end{pmatrix}, \quad C = \begin{pmatrix} c_{11} \\ c_{10} \\ c_{01} \\ c_{00} \\ \end{pmatrix}, \quad D = \begin{pmatrix} d_{11} \\ d_{10} \\ d_{01} \\ d_{00} \\ \end{pmatrix}, \end{aligned}$$

where $a_i, \ c_{ij}, \ d_{ij} \in \{0,1\}$ $\forall \ i,j \in \{0, 1\}$. The action rules determine how the individual will behave when it is chosen as a donor and meets a recipient it thinks to be good ($a_1$) or bad ($a_0$). For example, $a_1=1$ means to cooperate with someone good and $a_0=0$ to defect with someone bad. The assessment rules determine how the individual updates its opinions when it observes an interaction between two other players. The action of the donor and the current opinions of the observing individual (toward the two observed players) are considered. For example, the rule $c_{10}=1$ means that a good donor cooperating with a bad recipient is regarded as good afterwards, whereas $d_{01} = 0$ means that a bad donor defecting with a good recipient is regarded as bad afterwards. Assessment rules are divided into two here for simplicity (*C* applies to the case where donor cooperates and *D* applies to the case where donor defects). The opinion about the recipient is not updated. Errors may occur during assessment or while implementing an action. Following literature^25^, we consider (1) execution errors, at a rate $\mu _e$, where an individual does the opposite of what it intended (i.e. determined by her strategy) and (2) assessment errors, at a rate $\mu _a$, where an individual assigns the opposite opinion of what her assessment rules would suggest.

A large number of strategies are possible, and each strategy can differ in its evolutionary success. We want to compute the evolutionary success of different strategies and see if particular strategies stand out. Our method consists in deriving the long-run average proportion of good opinions others have on an individual, i.e. the individual’s h-score. We use this h-score to calculate the frequency of cooperation from and towards this individual which determinate its fitness. We apply this method to compare the h-score and the fitness of individuals in a population with a resident strategy and a single mutant strategy to perform an ESS analysis.

## Results

### Monomorphic population

We confirm that the analytical model correctly approximates the h-score and the probability of cooperation at equilibrium, by comparing the predictions of the analytical model with agent-based simulations for any possible strategies in a monomorphic population. Some pairs of strategies are equivalent (as formally defined in the mirror image section of the extended method). They simply assess and act upon opinions in an opposite way (what one would call good is called bad by the other). After keeping one instance of each pair, we are left with 258 strategies including 256 discriminator strategies, unconditional cooperator and unconditional defector. The simulations implement the aforementioned model with a population of 100 individuals and one observer per interaction. The results of the simulations are taken after $4\times 10^5$ interactions, and averaged over $10^5$ interactions and 30 independent replicates. We run analysis considering that (1) the error rate is negligible and (2) the error rate is not negligible. In the former, we consider that the error rate is equal to 0 in the analytical model and we keep a very low error rate in simulation (namely, $10^{-4}$). In the latter, we do not vary independently the execution and assessment error rates because we are interested in testing the robustness of the conclusion obtained from the model without errors, rather than describing the effect of a particular type of error.

The results show that the analytical model well approximates the h-score and the probability of cooperation at equilibrium. The mean difference between the h-score predicted and simulated is 0.014 in absence of error and 0.005 in presence of error. The mean difference between the frequency of cooperation predicted and simulated is 0.01 in absence of error and 0.0009 in presence of error. The detailed results are provided in SI. The results of the simulations are illustrated in supplementary Figure S1 and summarised in supplementary Figure S2.

### Evolutionary invasion analysis

We now use the analytical model to conduct an evolutionary invasion analysis (in short, ESS analysis). As common assumptions in ESS analysis^26^, we assume that (1) mutations are rare and thus, there is at most one mutant strategy *m* at a time in a population of individuals with resident strategy *r*, (2) the mutant’s effect is negligible on the dynamics and (3) population size is large enough so that stochasticity in selection is negligible. To know if a strategy can be invaded or not by another, we compute the difference of absolute fitness between a mutant strategy in a population of resident strategy. If the fitness of the mutant is lower or strictly equal, the mutant disappears and the resident resists invasion. If a strategy resists invasion from all other possible strategies, it is an ESS. Unlike the previous section, fitness and h-score are now directly computed rather than simulated. We consider that any differences in fitness less than $10^{-4}$ are equal to 0 because of the floating point error.

#### In absence of errors

First, we consider that the errors are negligible, that is $\mu _a = 0$ and $\mu _e = 0$.

**Figure 1:**
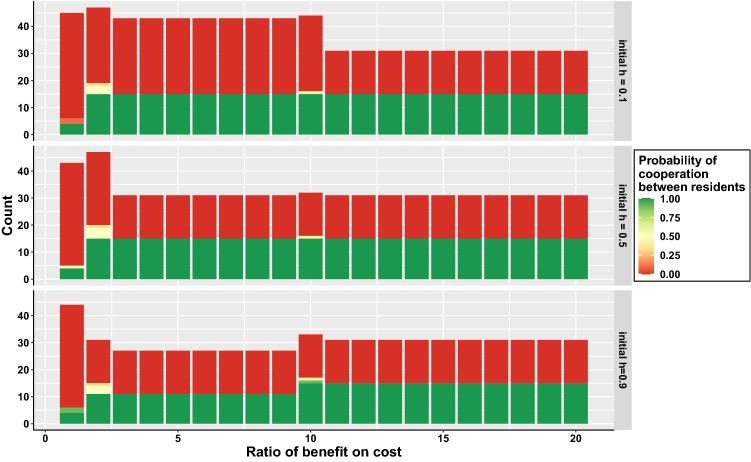
Number of ESS as a function of the benefit to cost ratio, *b*/*c*. The colour represents the probability of cooperation between residents. The results are presented for different initial h-scores *h*(*t*0) (0.1, 0.5 and 0.9, in top, middle and bottom rows, respectively).

Figure 1 shows that there are multiple evolutionary stable strategies (ESS), that is strategies that can not be invaded by others. Supplementary Figure S3 shows that some strategies are not ESS for all studied initial h-score *h*(*t*0). We focus on strategies that are ESS for all three initial h-scores.

**Figure 2:**
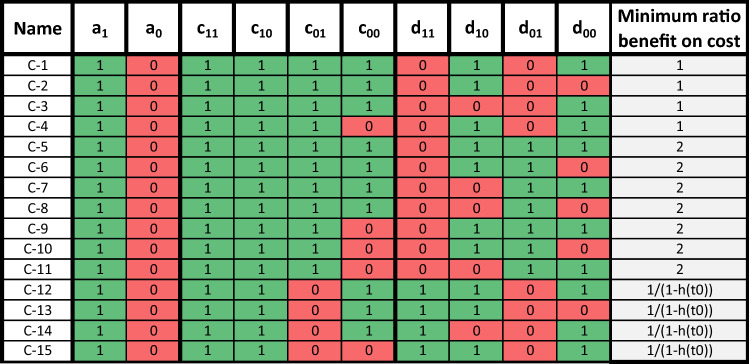
List of strategies that are cooperators and ESS for any initial h-score and at least one value of the benefit to cost ratio, *b*/*c*. The last column represents the minimum ratio for which the strategy is ESS for any initial h-score.

The ESS can be divided in two groups, strategies which cooperate and avoid exploitation, and those which defect and efficiently exploit others. There are strategies that have an intermediary probability of cooperation but they are only ESS for a specific benefit to cost ratio so we do not discuss them further here. We present the 15 strategies that are ESS and cooperators in Fig. 2, with the minimum benefit to cost ratio required for a strategy to be an ESS. We present the 38 strategies that are defectors in supplementary Figure S4, with the maximum benefit to cost ratio required for a strategy to be ESS. We call the ESS cooperator and defector strategies respectively C-* and D-*. We name each ESS cooperator, with C1 representing the first ESS cooperator in the table, C2 the second, and so on.

**Figure 3:**
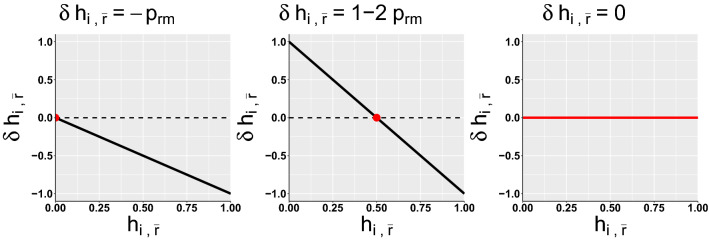
Differential equation of h-score of resident on mutant for the three types of ESS cooperators when mutant is always defect (AllD). From left to right, strategies that are ESS for a ratio of 1, 2 and $\frac{1}{1-h(t0)}$.

First, we look closely at the ESS cooperators. In term of behaviours, a distinctive feature of the ESS cooperators is that they fully cooperate with each other while cooperating less with mutant defectors. By cooperating with each other, they sustain the highest possible fitness for cooperators. By cooperating less with mutant defectors, they limit the fitness of the mutant to be less than or equal to their fitness, providing that the benefit of cooperation is high enough.

First, all the ESS cooperators have $c_{11} = 1$ and $c_{10} = 1$. It means that they consider that cooperation from an individual seen as good is always rewarded by future cooperation. Ultimately, this results in a population of individuals which see each other as good and always cooperate with each other. This allows the strategies to maintain cooperation once established.

Second, the ESS cooperators have either $d_{11} = 0$ or $d_{01} = 0$, or both. This means that they will consider individuals defecting towards good individuals as (partly or totally) bad. Because the ESS cooperators consider each other as good once h-score of 1 is reached, this allows ESS cooperators to defect with individuals that defect with them. ESS cooperators differ in their capacity to efficiently reciprocate defection. First, there are strategies that have both $d_{11} = 0$ and $d_{01} = 0$. They are ESS on the whole range of benefit to cost ratios (C1-C4). It is because they have an average opinion of 0 on defectors, and thus will always defect with them, as shown in the left panel of Fig. 3. Second, there are strategies that have both $d_{11} = 0$ and $d_{01} = 1$, which are ESS if the benefit is at least twice larger than the cost (C5–11). These strategies have half of the time good opinion (and cooperating) with mutant defectors, and half of the time bad opinion (and defecting) with mutant defectors. Finally, there are strategies which have $d_{11} = 1$ and $d_{01} = 0$ (C12–15) and for which their evolutionary stability depends of the initial h-score. For instance, they are ESS for a ratio $b/c > 10$ if the initial h-score is 0.9 or ESS for a ratio $b/c > 2$ if the initial h-score is 0.5. These strategies have in common that their opinions of mutant defecting with good individuals remain roughly the same. For instance, one strategy gives 0 to bad individuals defecting and gives 1 to good individuals defecting. Thus, the frequency of cooperation received by mutant defectors is approximately the initial h-score *h*(*t*0) and their fitness *h*(*t*0)*b*. Strategies C12–15 can not be invaded by a defector when their fitness $b-c > h(t0)b$. This equation can be rearranged, leading to $b/c > 1/(1-h(t0))$.

To summarise, the rules of ESS cooperators make them efficiently reciprocate once cooperation is fully established. The first pattern maintains cooperation while the second makes them defect with mutant defectors. Yet, this does not assure that they reach cooperation in the first place. For that, we can observe that ESS cooperators, besides $c_{11} = 1$ and $c_{10} = 1$, judge a number of other encounters as good. This number and the type of encounters can vary but they judge enough cases as good so that the h-score of an individual with a resident strategy seen by other individuals with the same strategy, increases towards 1 (the differential equation is always positive). This ensures that they go towards full cooperation even in presence of initial disagreement. For instance, the first three strategies (C1–C3) consider that cooperating is good and at least one other case as good. Because these strategies cooperate with a probability *h*, it ensures that the differential equation is always positive $h + p(d_{?0}) - h \ge 0$. Another example is C4, which might appear surprising as they consider one cooperation as bad $c_{00} = 0$. However, this case is very rare and it leads to the differential equation remaining positive.

In short, the strategies that are evolutionary stable and cooperators have rules that (1) establish full cooperation with each other, (2) sustain full cooperation when established, and (3) reduce the frequency of cooperation with mutant defectors. Note that the presence of a single of these features in a strategy does not mean that the strategy will be ESS. Indeed, we looked at the common patterns among the ESS rather than correlating the rules with the success of strategies. Finally, we observe a diversity of rules because first, strategies can differ in their capacity to defect with defectors, and second, different rules can lead a population to full cooperation on the long term.

We now look at defector strategies that are ESS. Again, there are numerous strategies fulfilling these conditions but they have similar behaviour. Their distinctive feature is that they have a lower probability to cooperate with the mutant, than the mutant have with the resident. The rules have in common the pattern that $d_{10} = d_{00} = 0$. In other words, they always defect with individuals defecting with individuals they see as bad. This allows them to avoid cooperation when individuals do not cooperate with them. Again, defectors can be separated into different types as a function of the maximum ratio of benefit to cost required for them to be ESS. First, the defectors that are ESS for the whole range of benefit studied (D1–D16) never cooperate with each other nor with the mutant. They are behaviourally equivalent to strategies that always defect. This means that they do not pay any cost and thus no strategies can have a higher fitness than them. They also all have in common that $c_{10} = c_{00} = 0$. This means that any strategy interacting with them (that they consider as bad) will be considered bad, and receive future defection. Second, some defectors are ESS only for a very limited range of the benefit to cost ratio. These strategies cooperate with mutant, but at a lesser rate than mutant with resident. This means that mutant can invade if the benefit they received by cooperation outweighs the cost of their cooperation. Without going into the details, these strategies do not have the rules of $c_{10} = c_{00} = 0$ as previous strategies, and thus are not perfect defectors.

We notice that there are a large number of cases of polymorphism among these strategies. In the cases where these strategies can be invaded, there are between 75.5 and 88.4% of cases with polymorphism against 5–17% when looking at any strategies. The reason is that if the benefit that mutant provides to resident is negligible when mutant are a minority, it is not the case anymore when they compose most of the population. Thus, defector strategies that are ESS for only a limited set of the benefit to cost ratios could still be frequent for other ratios.

#### In presence of errors

**Figure 4:**
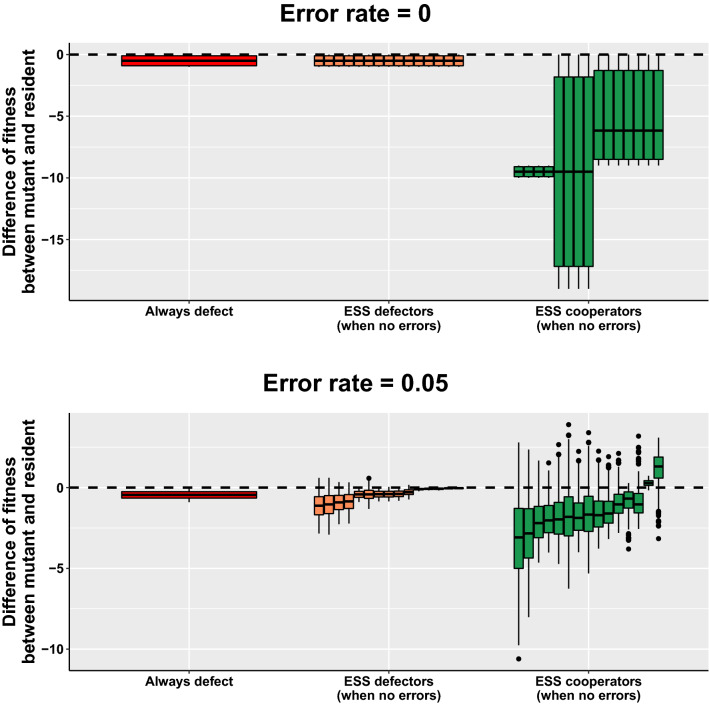
Difference of fitness between mutant and resident $w_m - w_r$, for different strategies that are ESS when there are no errors. We differentiate between strategies that were cooperators, defectors and the strategy that always defect (AllD), which is the only ESS in presence of errors. The results are presented for a high benefit to cost ratio ($b/c = 20$) to highlight the difference of fitness. Results for other, smaller benefit to cost ratios, can be found in supplementary figures S5 and S6, showing the same trend.

When the errors are not negligible, the previously identified ESS are not evolutionary stable anymore except for always defect (AllD). This is because the errors in assessment lead discriminating cooperators to cooperate less, and discriminating defectors to defect less. For instance, the previously ESS cooperators maintain a lower level of cooperation between each other (and thus are easier to invade). In addition, the errors create disagreement and can have an effect on the long term. As a result, C-* can cooperate more with mutant than with themselves, even when mutant are strong cooperators e.g. judge good all except $d_{00}$. This is because the cooperation of such strategy breaks down only in specific cases, which allow them to quickly get their h-score back to 1. AllD is still an ESS even if it sometimes cooperates by mistake, because it is not affected by assessment errors and thus has still the lowest frequency of cooperation possible.

To gain further insights, we look at the difference of fitness between mutant and resident for different resident strategies. This difference of fitness gives us hints on the success of strategies when evolution is stochastic, that is when invasion is a probability based on the difference of fitness. We show the results for a high benefit to cost ratio in Fig.  4 and for other ratios in supplementary Figures S5 and S6. First, Fig. 4 shows that in absence of error, ESS cooperators have a higher difference of fitness as the benefit increases. This result suggests that ESS cooperators could be more prevalent than ESS defectors when selection is weak and if the benefit of cooperation is sufficiently high. Second, Fig. 4 shows the same (but weaker) trend when errors are not negligible. In particular, the difference of fitness is higher for C15 than the only ESS with error: always defect against the majority of mutants. This suggests that if in presence of errors, C15 is not ESS anymore, it could still be a very frequent strategy (and more frequent than always defect).

## Discussion

Among the large number of possible moral rules, previous work shows that only a few of them stand out and should be observed in the real world^11^. Yet, models studying the evolution of moral rules considered either public assessment or a limited number of strategies and it still lacks of an exhaustive evolutionary analysis of moral rules with private assessment. In this paper, we fill this gap by building an analytical model to describe the change in opinions as a function of time. We used this model to study the invasibility of any strategies by any other strategies up to third-order assessment rules, and identify the evolutionary stable strategies. Our main results can be summarised in three points.

First, previous results suggested that considering private information breaks down cooperation and limits the evolution of cooperative moral rules by creating disagreement^27^. Our results show that this conclusion does not hold when interactions are long enough. In this case, our results show that there are evolutionary stable rules implementing cooperation even when assessment is private. This result is explained by the fact that some rules are capable of suppressing disagreement on the long term. In addition, our results show that the number of ESS in our study can even be higher than the number of ESS previously found when considering public information^11^. This is because multiple rules can end up implementing the same frequency of cooperation on the long term. For instance, strategies C-1 and C-3 differ in their rules about good individuals defecting with bad individuals ($d_{10}$) but they still end up with full cooperation at equilibrium. This adds long-term interactions to the list of mechanisms which can explain the evolution of indirect reciprocity when assessment is private, alongside empathy^20^, pleasing^28^ and public institutions^21^.

Second, we identify the important properties of the rules that are ESS and cooperators. These strategies consider (1) good cooperators as good, (2) all or a part of defectors towards good individuals as bad, and (3) a varying number of other cases as good. The last rule allows the strategy to converge toward a full population of good cooperators, and the first two rules allow them to efficiently reciprocate once good reputation is established.

**Table 1:** Presentation of the main strategies identified in the literature, i.e. image scoring, standing strategy and the leading eight; and the ESS cooperators identified in our analysis C1–15. * mark wildcards.

	$c_{11}$	$c_{10}$	$c_{01}$	$c_{00}$	$d_{11}$	$d_{10}$	$d_{01}$	$d_{00}$
Image scoring	1	1	1	1	0	0	0	0
Standing strategy	1	1	1	1	0	1	0	1
Leading 1-8	1	*	1	*	0	1	0	*
C1–4	1	1	1	*	0	*	0	*
C5–11	1	1	1	*	0	*	1	*
C12–15	1	1	0	*	1	*	0	*

Note that all C1–15 have an additional restriction: $p(o,r,r) > h_{r,\bar{r}}$ , which ensures that the h-score $h_{r,\bar{r}}$ increases up to 1 (see Eq. 4). This means that a maximum of one of the wildcards can be equal to 0 for C1–4 and C12–15, and a maximum of two wildcards can be equal to 0 for C5–11.

How do these successful rules compare to rules previously identified in the literature? To answer this question, we present the main rules in the comparative Table 1. A significant part of previous work has focused on two famous strategies, image scoring^4^, that is cooperate with cooperators and defect with defectors, and standing^10,29^, that is cooperate with cooperators, cooperate with defectors towards defectors and defect with defector towards cooperators (see Table 1). Image scoring was historically one of the first strategies to successfully implement indirect reciprocity^4^, but later work showed that standing is more evolutionary successful^10^. Our results concur as we found the standing strategy to be evolutionary stable for any benefit superior to cost (standing strategy is C1), while image scoring is not an ESS. Note that image scoring would be an ESS if initial h-score is exactly 1.

In addition, our results show that the important rules of the standing strategy are “cooperate with good cooperators” and “defect with defectors against good”, and that the part “cooperate with defectors against bad” can vary. This distinction provides important new insights into the ongoing debate. Indeed, some experimental evidence supports the presence of standing strategy in natural populations^30^ while others^31^ appear to be against. In laboratory experiments^31^, researchers compared the amount of defection received by an unconditional defectors and a “justified” defectors, that is individuals that defect with previous defectors. Their results showed that the difference is not strong enough to support standing strategy. As pointed out^10^, these conclusions could be limited as they do not measure the amount of defection received by individuals that refuse to help previous donors. Our results show rigorously here that it is this amount of defection towards defectors against good individuals that matters for the success of the standing strategy rather than the cooperation toward justified defectors as measured in the experiments. Thus, our results suggest that further experiments with different measures is required to reject or accept the prevalence of standing strategy.

Our work also follows the exhaustive evolutionary analysis which showed eight successful strategies (called leading eight^11^). A direct comparison of the leading eight and the ESS described here is limited because this previous study focused on the evolutionary success of different action rules, i.e. what is the best action rule for a given assessment rule, while the model presented here focuses on assessment rules, i.e. how to judge someone. Yet, it can shed light on main differences between private and public assessment. First, the C-* strategies require that $c_{10}=1$, a rule which is shared by only half of the leading eight. This rule is crucial with private assessment to avoid cooperators loosing their good standing. Those leading eight strategies which do not share this rule were shown to suffer greatly by private assessments before^27^. Second, C*-rules also require that enough cases are considered good so that the whole population converge towards being good cooperators. On the other hand, C-* rules can vary in cases where leading eight can not. For instance, the leading eight always consider defection towards a good individual as bad. This is shared by the most successful strategies found here. However, the C-* strategies can also consider defection in one situation ($d_{11}$ or $d_{01}$) as good and still be ESS given that the benefit of cooperation is high enough. This is because such a rule in public assessment would lead to all individuals cooperating with defectors while this happens partially with private assessment.

Third, we find that the presence of errors breaks down the evolutionary stability of the previously identified strategies. This is because the property of these rules that allow them to converge toward full cooperation, also makes them vulnerable to errors. This result suggests that private assessment rules could not evolve when errors are frequent, and that public assessment for instance supported by an institution could be preferred in this case^21^. However, this result is mitigated by two points. First, we have considered that any difference of fitness, however small, leads to invasion or not. This is a classic assumption of ESS analyses but in the real world, selection can be weaker and stochasticity can result in non-ESS to be frequent. Important first steps have been made by a recent paper which considered stochastic evolution of a population mixing one discriminator strategy, with unconditional cooperators and defectors^22^. An extension could consider a population of different discriminator strategies in co-presence. Second, the effect of errors could be suppressed by additional mechanisms. Evidence shows that not only the outcome of an action plays a role in assessments but also the intention behind this action^32,33^, and thus errors in actions could have a limited effect. Another example is the role of communication and conformity which could counterbalanced the effect of errors and drive the h-score towards a general agreement. Further work integrating these mechanisms would provide a more realistic model and test if the strategies identified here could be frequent in presence of errors.

Results from models of indirect reciprocity can be confronted to the donor game conducted in laboratory experiments. For instance, experiments conducted by^34^ showed that information about the partners’ previous partners’ reputation increases the level of cooperation. This is in agreement with our results that all the C-* use second-order information. Second, recent experiments have studied the strategies employed by individuals^35^. They show that individuals often requested second-order information, and at a higher frequency when their partner has previously defected. We find some similarity in our results. All the C-* require to know the past interactions of their partner to judge its action when the partner defected, while only eight strategies require this information when the partner cooperated. This goes to 3 against 1 if we considered the most successful strategies C1–4. Last but not least, they showed a strong variation in behavioural strategies. This is in line with ours results, which show that diverse behavioural strategies can be employed. However, these comparisons are limited as our model considers a large group size and long interactions, which are both assumptions often absent in laboratory experiments. A more promising path to test our results would be in study in natural populations.

We have made a number of assumptions in this model that need to be discussed. First, we approximated the reputation dynamics by a deterministic approach. This required two main assumptions, that the size of the population is infinitely large and that the number of observers is finite. The first assumption means that the results in this paper are applicable only to cases where the population is sufficiently large. The second assumption results naturally from physical limits of the number of observers, e.g. it is likely that an increase of ten fold of group size does not mean an increase of ten fold of the number of observers. However, it is important to note that there are possible exceptions, in particular systems where actions are widely shared e.g. e-commerce or medieval merchant guilds^36^.

Second,we have considered that the initial h-scores are the same for all individuals, including the resident and mutant strategies. In real world, the opinion of an individual on a newly met individual could be part of the individual strategy (in the same way that tit-for-tat could play cooperate or defect at the first round). We did not consider this here to keep the number of strategies reasonable and we focused on the strategies that are ESS for diverse initial h-scores. Future work could integrate the initial h-score in the strategy and replicate the evolutionary analysis.

To conclude, the contribution of this paper is two fold. First, it provides a first exhaustive evolutionary analysis of moral rules with private assessment. It provides more realistic results, as a large number of real-world situations (including most of laboratory experiments) includes private assessment. Second, it provides an analytical model that describes the opinion dynamics when assessment is private and allows further investigation of the issue accurately and at a faster speed than with simulations, enabling exhaustive analyses. The model can easily be extended to integrate other mechanisms. A natural progression of this work is (1) to study strategies up to second-order action rules where action also depends of actor’s reputation to compare results with the previous exhaustive evolutionary analysis with public assessment^11^, (2) to integrate the effect of communication and conformity^37^ as it plays a prevalent role in indirect reciprocity^24^ and can be easily integrated in the model^38^, and (3) to add the possibility of costly punishment which has been shown to interact with indirect reciprocity^39^.

## Method

We build a deterministic model that approximates the average fitness of an individual of a given strategy. We first consider a *monomorphic* population where all individuals have the same resident strategy. We do so to introduce the method in its simplest form. We consider that the number of interactions is large enough, and thus, the fitness $w_i^*$ of a focal individual *i* is its average payoff (*N* is the population size):

2 $$\begin{aligned} w_{i}^* = \frac{1}{N-1}\sum _{r = 1}^{N-1} \left( b p^*(c_{i,r}) - c p^*(c_{r,i})\right) . \end{aligned}$$

The fitness of an individual *i* is the benefit *b* received when other individuals cooperate with the individual *i*, discounted by the cost *c* paid when the focal individual *i* cooperates. The probability that an individual *r* cooperates with individual *i* is denoted by $p(c_{i,r})$. The superscript $^*$ denotes that the fitness and probability of cooperation considered are at equilibrium. This probability itself depends on the many opinions that individuals have on each other, which is difficult to track analytically. Instead of describing all the opinions, we define a *h-score* of an individual *i* as the proportion of other individuals with opinion 1 on *i*, or the average reputation of *i*.

The h-score is useful because considering that the number of individuals is large enough and that the donor, recipient and observers are chosen randomly, the h-score also describes the probability that a random individual has an opinion of 1 on the focal individual *i*, that is $h_{i, {\overline{r}}} = p(o_{ir} = 1)$. Thus, we can combine h-score and the action rules which describe how individuals act upon a given encounter, to describe the probability of cooperation:

3 $$\begin{aligned} p^*(c_{i,r}) = h_{i,r} a_1 + (1-h_{i,r})a_0. \end{aligned}$$

Similarly, using the assessment rules, we can calculate the probability that h-score increases or decreases after an interaction $p(o_{r,r,r})$, and thus, the dynamics of h-score over time. The formula is given in Equation 9 in the detailed method in **SI**. Execution and assessment errors modify respectively the probability of cooperation or the probability of h-score to increase or decrease after an interaction as described in the equations 12 and 13 of the extended method section (**SI**).

So far, we derived the change in h-score for an individual with a given strategy but we would like to derive the change in h-score for any individuals. Let us note that because individuals have the same strategy, the direction of change is similar across individuals and their h-score will converge towards the same equilibrium points. In addition, we make the assumption that the number of observers is small and independent of the population size. We also assume that the initial h-score of all individuals are the same. Following these two assumptions, the differences in h-score between individuals due to stochasticity is small and negligible on the dynamics. Because the change in h-score is very small after an interaction, it can be approximated by the following differential equation^40^ (see details in SI)

4 $$\begin{aligned} \frac{d(h_{r, {\overline{r}}})}{dt} = p(o_{r,r,r}) - h_{r, {\overline{r}}}. \end{aligned}$$

The average h-score at equilibrium can be found by solving the equation $\frac{d(h_{r, {\overline{r}}})}{dt} = 0$. This equation is a polynomial of $h_{r, {\overline{r}}}$ of a maximum degree of three (see Equations 19 to 22 in SI). The stability of equilibrium points is determined by looking at the sign of the derivative at the equilibrium points^26^.

We now extend the analytical model to conduct an evolutionary invasion analysis (in short, ESS analysis). To know if a strategy can be invaded or not by another, we need to compute the difference of absolute fitness between a mutant strategy in a population of resident strategy. If the fitness of the mutant is greater than that of the resident, the mutant invades the population and becomes resident. If the fitness of the mutant is lower, the mutant disappears and the resident resists invasion. When the two values of fitness are equal, the resident also resists invasion because in an infinitely large population, a mutant strategy can not invade by drift. If two strategies can mutually invade, there will be polymorphism.

The difference of fitness between a mutant $w_m$ and a resident $w_r$ is given as follows:

5 $$\begin{aligned} \Delta w = w_m - w_r = p^*(c_{m,r})b - p^*(c_{r,m})c - p^*(c_{r,r})(b-c). \end{aligned}$$

The fitness of the mutant is the benefit received when a resident cooperates with the mutant discounted by the cost of the cooperation from mutant to resident. There are three different probabilities of cooperation. The probability of cooperation between residents $p(c_{r,r})$ is calculated as in the case of a monomorphic population. The two remaining probabilities of cooperation can be computed as previously using h-score and action rules (Equation 15 in SI). To find the probability of cooperation at equilibrium, we describe the dynamics of the h-score as previously

6 $$\begin{aligned} \begin{aligned} \frac{d(h_{m,{\overline{r}}})}{dt}&= p(o_{m,r,r}) - h_{m,{\overline{r}}},\\ \frac{d(h_{{\overline{r}},m})}{dt}&= p(o_{r,r,m}) - h_{{\overline{r}},m}. \end{aligned} \end{aligned}$$

The probabilities of h-score to increase after the observation of an interaction, *p*(*o*), can be described using the h-score and the assessment rules as previously (Equation 17 in SI). This system of two polynomial equations with two unknowns are solved numerically. To determinate the stability of the equilibrium points, we look at the Jacobian matrix at the equilibrium of interest. The equilibrium is locally stable if the real part of the leading eigenvalue is negative^26^. Errors are integrated in the same way as in the case of monomorphic populations.

## Supplementary Information

Supplementary Information.

## Data Availability

The code is available online at ‘https://github.com/CedricPerret’ in the project ‘RepDyn’.

## References

[1] Hamlin JK, Wynn K, Bloom P, Mahajan N (2011). How infants and toddlers react to antisocial others. Proc. Natl. Acad. Sci. USA.

[2] Harms W, Skyrms B (2009). The Oxford Handbook of Philosophy of Biology.

[3] Alexander RD (1987). The Biology of Moral Systems (ed Routledge).

[4] Nowak MA, Sigmund K (1998). Evolution of indirect reciprocity by image scoring. Nature.

[5] Brandt H, Ohtsuki H, Iwasa Y, Sigmund K (2007). Mathematics for Ecology and Environmental Sciences.

[6] Nowak MA, Sigmund K (2005). Evolution of indirect reciprocity. Nature.

[7] Axelrod R, Hamilton WD (1981). The evolution of cooperation. Evolution.

[8] Hilbe C, Martinez-Vaquero LA, Chatterjee K, Nowak MA (2017). Memory- n strategies of direct reciprocity. Proc. Natl. Acad. Sci. USA.

[9] Dunbar RI (1998). The social brain hypothesis. Evol. Anthropol. Issues News Rev..

[10] Leimar O, Hammerstein P (2001). Evolution of cooperation through indirect reciprocity. Proc. R. Soc. B Biol. Sci..

[11] Ohtsuki H, Iwasa Y (2004). How should we define goodness?—Reputation dynamics in indirect reciprocity. J. Theor. Biol..

[12] Olson KR, Spelke ES (2008). Foundations of cooperation in young children. Cognition.

[13] Kenward B, Dahl M (2011). Preschoolers distribute scarce resources according to the moral valence of recipients’ previous actions. Dev. Psychol..

[14] Meristo M, Surian L (2013). Do infants detect indirect reciprocity?. Cognition.

[15] Hamlin JK, Wynn K, Bloom P (2007). Social evaluation by preverbal infants. Nature.

[16] Smith KM, Larroucau T, Mabulla IA, Apicella CL (2018). Hunter- gatherers maintain assortativity in cooperation despite high levels of residential change and mixing. Curr. Biol..

[17] Smith KM, Apicella CL (2020). Hadza hunter-gatherers disagree on perceptions of moral character. Soc. Psychol. Personal. Sci..

[18] Okada I, Sasaki T, Nakai Y (2018). A solution for private assessment in indirect reciprocity using solitary observation. J. Theor. Biol..

[19] Uchida S (2010). Effect of private information on indirect reciprocity. Phys. Rev. E.

[20] Radzvilavicius AL, Stewart AJ, Plotkin JB (2019). Evolution of empathetic moral evaluation. Elife.

[21] Radzvilavicius AL, Kessinger TA, Plotkin JB (2021). Adherence to public institutions that foster cooperation. Nat. Commun..

[22] Okada I (2020). Two ways to overcome the three social dilemmas of indirect reciprocity. Sci. Rep..

[23] Okada I (2020). A review of theoretical studies on indirect reciprocity. Games.

[24] Dunbar R (1998). Grooming, Gossip, and the Evolution of Language.

[25] Santos FC, Santos FP, Pacheco JM (2018). Social norm complexity and past reputations in the evolution of cooperation. Nature.

[26] Otto SP, Day T (2007). A Biologist’s Guide to Mathematical Modeling in Ecology and Evolution.

[27] Hilbe C, Schmid L, Tkadlec J, Chatterjee K, Nowak MA (2018). Indirect reciprocity with private, noisy, and incomplete information. Proc. Natl. Acad. Sci. USA.

[28] Krellner M, Han TA (2021). Pleasing enhances indirect reciprocity-based cooperation under private assessment. Artif. Life.

[29] Sugden R (1986). The Economics of Rights, Co-operation and Welfare.

[30] Hilbe C, Šimsa Š, Chatterjee K, Nowak MA (2018). Evolution of cooperation in stochastic games. Nature.

[31] Milinski M, Semmann D, Bakker TC, Krambeck HJ (2001). Cooperation through indirect reciprocity: Image scoring or standing strategy?. Proc. R. Soc. B Biol. Sci..

[32] Han TA (2013). Intention Recognition, Commitment and Their Roles in the Evolution of Cooperation: From Artificial Intelligence Techniques to Evolutionary Game Theory Models.

[33] Barrett HC (2016). Small-scale societies exhibit fundamental variation in the role of intentions in moral judgment. Proc. Natl. Acad. Sci. USA.

[34] Bolton GE, Katok E, Ockenfels A (2005). Cooperation among strangers with limited information about reputation. J. Public Econ..

[35] Swakman V, Molleman L, Ule A, Egas M (2016). Reputation-based cooperation: Empirical evidence for behavioral strategies. Evol. Hum. Behav..

[36] Greif A (2006). Institutions and the Path to the Modern Economy: Lessons from Medieval Trade.

[37] Cavalli-Sforza LL, Feldman MW, Chen KH, Dornbusch SM (1982). Theory and observation in cultural transmission. Science.

[38] Denton KK, Ram Y, Liberman U, Feldman MW (2020). Cultural evolution of conformity and anticonformity. Proc. Natl. Acad. Sci. USA.

[39] Hilbe C, Traulsen A (2012). Emergence of responsible sanctions without second order free riders, antisocial punishment or spite. Sci. Rep..

[40] Bortolussi L, Hillston J, Latella D, Massink M (2013). Continuous approximation of collective system behaviour: A tutorial. Perform. Eval..

