# Supplementary material for: The evolution of moral rules in a model of indirect reciprocity with private assessment
Source: Sci Rep. 2021 Dec 8;11:23581. doi: 10.1038/s41598-021-02677-2 (PMC8654852; doi:10.1038/s41598-021-02677-2)
Supplement: Supplementary file 1 — Supplementary Information. [file 41598_2021_2677_MOESM1_ESM.pdf]

Supplementary Information for

The evolution of moral rules in a model of indirect reciprocity with private assessment

Cedric Perret[a,b,*], Marcus Krellner[a], and The Anh Han[a]

[a]Teesside University, Southfield Rd, Middlesbrough TS1 3BX, United Kingdom
[b]University of Exeter, Penryn TR10 9FE, United Kingdom
[*]Corresponding author

Detailed methods

We build a deterministic model that approximates the average fitness of an individual of a given strategy. We first consider a monomorphic population where all individuals have the same resident strategy.

We denote i a focal individual, r a random individual other than the focal, and N the population size. Considering that the number of interactions is large enough, the fitness w_i^* of i is its average payoff

$$w_i^* = \frac{1}{N-1} \sum_{r=1}^{N-1} \left(bp^*(c_{i,r}) - cp^*(c_{r,i}) \right). \tag{1}$$

The fitness of an individual i is the benefit b received when other individuals cooperate with i, discounted by the cost c when i cooperates. The probability that an individual r cooperates with individual i is denoted by $p(c_{i,r})$. The superscript * denotes that the fitness and probability of cooperation are considered at equilibrium. The probability of cooperation is given by:

$$p^*(c_{i,r}) = p^*(o_{ir} = 1)a_1 + p^*(o_{ir} = 0)a_0, \tag{2}$$

where o_{ir} stands for the opinion of individual r toward individual i. The probability of cooperation is the sum of the probabilities of encounters where the action rules dictate cooperation. To simplify the writing, we define a probability vector M which describes the probability of each encounter.

$$M^{p(o_{jr})} = \begin{pmatrix} p(o_{jr} = 1) \\ p(o_{jr} = 0) \end{pmatrix} = \begin{pmatrix} p(o_{jr} = 1) \\ 1 - p(o_{jr} = 1) \end{pmatrix}, \tag{3}$$

$$M^{(p(o_{ir}),p(o_{jr}))} = \begin{pmatrix} p(o_{ir} = 1, o_{jr} = 1) \\ p(o_{ir} = 1, o_{jr} = 0) \\ p(o_{ir} = 0, o_{jr} = 1) \\ p(o_{ir} = 0, o_{jr} = 0) \end{pmatrix} = \begin{pmatrix} p(o_{ir} = 1)p(o_{jr} = 1) \\ p(o_{ir} = 1)[1 - p(o_{jr} = 1)] \\ [1 - p(o_{ir} = 1)]p(o_{jr} = 1) \\ [1 - p(o_{ir} = 1)][1 - p(o_{jr} = 1)] \end{pmatrix}. \tag{4}$$

This vector contains two probabilities for action rules, that are the probabilities that a recipient j is seen as 1, $p(o_{jr} = 1)$, or 0, $p(o_{jr} = 0)$. Note that the probability that the recipient is seen as 0 is the probability that this recipient is not seen as 1, that is $p(o_{jr} = 0) = 1 - p(o_{jr} = 1)$. The probability vector contains four probabilities for assessment rules, that are the probabilities that a donor i and recipient j are respectively seen as 11, $p(o_{ir} = 1, o_{jr} = 1)$, 10, $p(o_{ir} = 1, o_{jr} = 0)$, 01, $p(o_{ir} = 0, o_{jr} = 1)$, or 00, $p(o_{ir} = 0, o_{jr} = 0)$. These probabilities can be described by a product of probabilities. For instance, the probability that both a donor and recipient are seen as 1 is equivalent to the probability that a donor is seen as 1 times the probabilities that the recipient is seen as 1.

Because the donor, recipient and observer are chosen randomly, these probabilities are described by the proportion of individuals with an opinion of 1 on the individual i. We define a *h-score* of an individual i as

$$h_{i,\bar{r}} = \frac{1}{N-1} \sum_{r=1}^{N-1} o_{ir}. \tag{5}$$

The h-score, $h_{i,\bar{r}}$, of a focal individual i is the proportion of other individuals with opinion 1 on the focal individual, or the average reputation of i. Similarly, the average opinion i has about other players can be written as

$$v_{\bar{r},i} = \frac{1}{N-1} \sum_{r=1}^{N-1} o_{ri}. \tag{6}$$

The h-score is useful because considering that the number of individuals is large

enough and that the donor, recipient and observers are chosen randomly, it describes the probability that a random individual has an opinion of 1 on i. In other words, $h_{i,\overline{r}} = p(o_{ir} = 1)$.

We now rewrite the probability of cooperation (see the definition of A in the main text)

$$p^*(c_{i,r}) = M^{(h^*_{i,\overline{r}})} A. \tag{7}$$

The fitness and the probability of cooperation depend of the h-score at equilibrium reached after a large number of interactions. In order to calculate it, we now describe the dynamics of opinions.

Opinion dynamics and h-score

To compute the h-score of an individual at equilibrium, we first describe how the h-score changes after one interaction. After an interaction where the focal individual i is the donor, her h-score will update to

$$\begin{aligned} h_{i,\overline{r}}(t+1) &= \frac{N - N_o}{N} h_{i,\overline{r}}(t) + \frac{N_o}{N} p(o_{i,r,r'}) \\ &= h_{i,\overline{r}}(t) + \frac{N_o}{N}[p(o_{i,r,r'}) - h_{i,\overline{r}}(t)], \end{aligned} \tag{8}$$

where N_o represents the number of observers of an interaction. A proportion $\frac{N-N_o}{N}$ of individuals keep the same opinions $h_{i,\overline{r}}(t)$, while the remaining proportion, $\frac{N_o}{N}$, observe the interaction and update their opinions. Observers update their opinions to 1 with a probability $p(o_{i,r,r'})$ with the indices representing respectively the donor, the recipient and the observer. It represents the probability that a random individual from the population judging the interaction of the focal individual i with another random individual r as 1. It is calculated in the same way as the probability of cooperation by summing the probabilities of encounters where an observer would assign 1, as follows (see the main text for the definitions of vectors C and D)

$$p(o_{i,r,r'}) = AM^{(h_{r,\overline{r}})} M^{(h_{i\overline{r}}, h_{r\overline{r}})} C + (1 - AM^{(h_{r,\overline{r}})}) M^{(h_{i\overline{r}}, h_{r,\overline{r}})} D. \tag{9}$$

It is the sum of the probability of encounters where the donor cooperates AM or not $1 - AM$, and an observer assigns 1 to the donor for this action. This probability depends of the h-score of other individuals, which might differ. However, the probability $p(o)$ is the same function of h-score for all individuals sharing

similar assessment rules and action rules. Thus, the h-score of individuals of the same strategy will follow the same dynamics and $h_{i,\overline{r}} = h_{r,\overline{r}} + \epsilon$ with the difference ϵ will only arise from stochasticity.

First, we make the assumption that the number of observers is small and independent of the population size. Second, we make the assumption that the initial h-scores are close, as done in previous work. Following these two assumptions, the difference due to stochasticity is small and negligible on the dynamics. Thus, we can consider that $h_{i,\overline{r}} = h_{r,\overline{r}}$. Second, because the amount of change in h-score is very small after an interaction, we can do a continuous approximation of the change in h-score and describe it by a differential equation [1]

$$\frac{d(h_{r,\overline{r}})}{dt} = p(o_{r,r,r'}) - h_{r,\overline{r}}. \tag{10}$$

The equilibrium points are the solutions of $\frac{d(h_{r,\overline{r}})}{dt} = 0$. The equation is a polynomial of maximum degree 3 which could be analytically solved (see below). Thus, there are at most three equilibrium points.

However, the solutions of a cubic polynomial equation can be in a complex form and therefore not informative. A lot of the terms will be vanished for a particular strategy (when A, C and D contain 0), it is easier to simplify the equation for each strategy and then do the analysis on the equation obtained.

The stability of equilibrium points is determined by looking at the sign of the derivative at the equilibrium points [2]. An equilibrium point $h^*_{r,\overline{r}}$ is stable if

$$\frac{d(\frac{d(h_{r,\overline{r}})}{dt})}{dh_{r,\overline{r}}}\Big|_{h_{r,\overline{r}}=h^*_{r,\overline{r}}} < 0. \tag{11}$$

Execution and assessment errors

So far, we considered that individuals never commit errors. Yet, more realistically, errors may occur during assessment or while implementing an action (i.e. cooperate or defect). As in [3], we consider (i) execution errors where an individual does the opposite of what it intended (i.e. determined by her strategy) and (ii) assessment errors where an individual assigns the opposite opinion of what her assessment rules would indicate.

Execution error. We consider that an execution error might happen after the donor has chosen its action at a rate μ_e. The presence of execution errors

modifies the probability of cooperation $p(c_{i,r})$ as follows

$$p(c_{i,r})|_{with\ error} = (1 - \mu_e)p(c_{i,r})|_{no\ error} + \mu_e(1 - p(c_{i,r})|_{no\ error}). \quad (12)$$

The probability of cooperation is the sum of (i) cases where the donor decided to cooperate and does not make an error and (ii) cases where the donor decided to defect and make an error.

Assessment error. We consider that an assessment error might happen after assessment at a rate μ_a. The presence of assessment errors modifies the probability $p(o_{i,r,r'})$ as follows

$$p(o_{i,r,r'})|_{with\ mutation} = (1-\mu_a)p(o_{i,r,r'})|_{no\ mutation}+\mu_a(1-p(o_{i,r,r'})|_{no\ mutation}). \quad (13)$$

The probability is the sum of (i) cases where there is no error and opinions are updated to 1 and (ii) cases where there is an error and the observer initially assigned 0 to the donor.

Evolutionary invasion analysis

We now model the evolutionary success of strategies using an ESS analysis. We need to compute the difference of absolute fitness between a mutant strategy in a population of resident strategy. The difference of fitness between that of a mutant, w_m, and that of a resident, w_r, is

$$\Delta w = w_m - w_r = p^*(c_{m,r})b - p^*(c_{r,m})c - p^*(c_{r,r})(b - c). \quad (14)$$

The fitness of the mutant is the benefit received when a resident cooperates with the mutant discounted by the cost of the cooperation from mutant to resident. There are three different probabilities of cooperation:

$$\begin{aligned} p^*(c_{m,r}) &= M^{(h^*_{m,\bar{r}})}A_r \\ p^*(c_{r,m}) &= M^{(h^*_{\bar{r},m})}A_m \\ p^*(c_{r,r}) &= M^{(h^*_{r,\bar{r}})}A_r \end{aligned} \quad (15)$$

The probability of cooperation and the h-score at equilibrium between residents are calculated in the previous section. To compute the probability of cooperation between mutant and resident, we need to describe the dynamics of the h-score

as previously

$$\frac{d(h_{m,\overline{r}})}{dt} = p(o_{m,r,r}) - h_{m,\overline{r}},$$
$$\frac{d(h_{\overline{r},m})}{dt} = p(o_{r,r,m}) - h_{\overline{r},m}, \tag{16}$$

where

$$p(o_{m,r,r}) = A_m M^{(h_{\overline{r},m})} M^{(h_{m,\overline{r}},h_{r,\overline{r}})} C_r + (1 - A_m M^{(h_{\overline{r},m})}) M^{(h_{m,\overline{r}},h_{r,\overline{r}})} D_r,$$
$$p(o_{r,r,m}) = A_r M^{(h_{r,\overline{r}})} M^{(h_{\overline{r},m},h_{\overline{r},m})} C_m + (1 - A_r M^{(h_{r,\overline{r}})}) M^{(h_{\overline{r},m},h_{\overline{r},m})} D_m. \tag{17}$$

This describes a system of two polynomial equations with two unknowns which are solved numerically. To determinate the stability of the equilibrium points, we look at the Jacobian matrix at the equilibrium of interest. The equilibrium is locally stable if the real part of the leading eigenvalue is negative [2]. Errors are integrated in the same way as in the case of monomorphic populations.

Mirror image

As described in reference [4], some pairs of strategies are equivalent. Formally, a strategy u is the mirror image of strategy v when

$$\begin{aligned}
a_{u1} &= a_{v0},\\
a_{u0} &= a_{v1},\\
c_{u11} &= 1 - c_{v00},\\
c_{u10} &= 1 - c_{v01},\\
c_{u01} &= 1 - c_{v10},\\
c_{u00} &= 1 - c_{v11},\\
d_{u11} &= 1 - d_{v00},\\
d_{u10} &= 1 - d_{v01},\\
d_{u01} &= 1 - d_{v10},\\
d_{u00} &= 1 - d_{v11}.
\end{aligned} \tag{18}$$

We keep only one of each of these mirror images for the analysis and we arbitrary decide to keep those strategies that cooperate if their opinion of the recipient is 1 (if it is thought to be good). Hence, the strategies use only three different action rules; the conditional 10 (cooperate with good but defect against bad)

and the two unconditional 00 (always defect) and 11 (always cooperate). Since the different assessment rules are irrelevant for cooperative behavior of these strategies, we keep only one instance of unconditional cooperator and defector (AllC and AllD), leaving us with 258 strategies to consider.

The realisable equilibrium points are the solutions in the definition domain of the reals in the interval $[0, 1]$. When there are no solutions in the definition domain, one of the boundary is the stable equilibrium point, 1 when the differential equation is positive and 0 when the differential equation is negative.

Extended results for monomorphic population

The results show that in absence of errors, 91.4% (236) of strategies have a single stable point, 5% (13) have two stable points and 3.5% (9) do not have a stable point. The last case occurs when the differential equation is equal to 0, and thus any point is an equilibrium point. An example is a strategy that assesses as good an individual that was previously good, and assess as bad an individual that was previously bad, as illustrated in Figure S1. We consider that the h-score is equal to the initial h-score when the whole domain is an equilibrium. This case never happens when errors are significant where 257 strategies have a single stable point and 1 has two stable points. In presence of multiple stable points, the h-score predicted depends of the initial conditions. When the initial h-score is exactly on the unstable point separating the two stable points, we consider that the h-score predicted is the average of the two stable points.

Finally, some strategies (30% in absence of error, none with errors) have a point which is neither stable or unstable. This case arises when the derivative of the differential equation is equal to 0 at the equilibrium. This means that the amount of change is slowly getting toward 0 as h-score get closer to the equilibrium point, as illustrated in Figure S1. We consider that eventually, the h-score will get close to the equilibrium point, and thus the predicted h-score is the equilibrium point.

Next, we compare the predictions of the analytical model with computational simulations. The results show that the analytical model correctly predicts the h-score (and thus the frequency of cooperation). Figure S2 shows that the difference between simulations and predictions are slightly higher when one of the dynamic particularity is present but it remains low.

Development of the differential equation

To find the h-score at equilibrium, we solve the differential equation $\frac{d(h_{r,\bar{r}})}{dt} = 0$, as presented in Equation 10. A general form of the equation can be found by developing the terms in Equation 9. Indeed, the equation reads (for simplicity, the h-score is noted as h)

$$\frac{d(h)}{dt} = \big[a_1 h + a_0(1-h)\big]\big[c_{11}h^2 + (c_{10}+c_{01})h(1-h) + c_{00}(1-h)^2\big] +$$
$$\big[1-(a_1 h + a_0(1-h))\big]\big[d_{11}h^2 + (d_{10}+d_{01})h(1-h) + d_{00}(1-h)^2\big] - h = 0. \tag{19}$$

The equation is a polynomial of maximum degree 3 of the form $xh^3 + yh^2 + zh + w = 0$, where

$$\begin{aligned}
&x = (a_1-a_0)(c_{11}-c_{10}-c_{01}+c_{00}) + (a_0-a_1)(d_{11}-d_{10}-d_{01}+d_{00}),\\
&y = (a_1-a_0)(c_{10}+c_{01}-2c_{00}) + a_0(c_{11}-c_{10}-c_{01}+c_{00}) +\\
&(a_0-a_1)(d_{10}+d_{01}-2d_{00}) + (1-a_0)(d_{11}-d_{10}-d_{01}+d_{00}),\\
&z = (a_1-a_0)(c_{00}) + (a_0-a_1)(d_{00}) + a_0(c_{10}+c_{01}-2c_{00}) + (1-a_0)(d_{10}+d_{01}-2d_{00}),\\
&w = a_0c_{00} + (1-a_0)d_{00},
\end{aligned} \tag{20}$$

which can be rewritten as

$$\begin{aligned}
&x = (a_1-a_0)(c_{11}-d_{11}-c_{10}+d_{10}-c_{01}+d_{01}+c_{00}-d_{00}),\\
&y = (a_1-a_0)(c_{10}-d_{10}+c_{01}-d_{01}-2c_{00}+2d_{00})\\
&\ +a_0(c_{11}-d_{11}-c_{10}+d_{10}-c_{01}+d_{01}+c_{00}-d_{00}) + (d_{11}-d_{01}-d_{10}+d_{00}),\\
&z = (a_1-a_0)(c_{00}-d_{00}) + a_0(c_{10}-d_{10}+c_{01}-d_{01}-2c_{00}+2d_{00}) + (d_{10}+d_{01}-2d_{00}) - 1,\\
&w = a_0(c_{00}-d_{00}) + d_{00}.
\end{aligned} \tag{21}$$

The derivative of the equation gives $3xh^2 + 2yh + z$. Thus, an equilibrium point $h^\star$ is stable if and only if

$$3xh^{\star 2} + 2yh^\star + z < 0. \tag{22}$$

Comparison between C-* and leading eight

All C-* share $c_{11} = 1$ and $c_{10} = 1$. The first rule, assessing the action as good when a good player cooperates with a good player, is also shared by all leading-eight. It seems to be an universal rule. The reason might be, that it is strictly required to reach an h-score of $r = 1$ (i.e. homogeneous good opinions) and therefore full cooperation. This is the case because the observation that c_{11} describes, becomes more and more frequent when r increases. It is more likely that someone good meets someone good when good opinions are more abundant. And with the universal action rules of discriminators, cooperation is also more likely for higher h-scores. Hence, if this assessment rule would state 'bad' instead ($c_{11} = 0$), this observation would lead to opinions turning bad, which would happen more often the more good opinions there are, opposing a negative feedback to an increase in h-score. (In fact, given that the equilibrium point is reached when turning bad is as likely as turning good, the maximum equilibrium h-score for strategies with $c_{11} = 0$ is for the strategy that assesses good for all other observations ($C = [0, 1, 1, 1], D = [1, 1, 1, 1]$) $r = 0.682$ (from $rrr = 1(1 - r)$).

The second rule that all C-* share, $c_{10} = 1$, is shared by only half of the leading-eight. Those leading eight which do not share this rule were shown to suffer greatly by private assessments before . It stands to reason, that it is necessary for private assessments to lessen the effect of disagreement.

All other of rules can go either way in the C-*, although there are systematic exceptions about combinations (see below). Not all C-* share the other universal rules of the leading eight: $c_{01} = 1$, $d_{11} = 0$, $d_{10} = 1$ and $d_{01} = 0$.

The C-* can be categorised into 3 groups. The first group, C1-4, contains 2 leading eight members. C1 is equivalent to L3 (also called simple-standing) and C4 is equal to L4. A third norm, C2 is equivalent to L1 (also called standing) in its assessment rules, but has slightly different action rules (The action rules of L1 are not possible under our framework). This group has five universal rules, 4 of which match all leading eight ($c_{01} = 1$, $d_{11} = 0$, $d_{10} = 1$ and $d_{01} = 0$, but $c_{10} = 1$ matches only half of them). The three wildcards (that can go either 1 or 0 in this group) follow an additional rule: at least 2 have to be good ($c_{00} + d_{10} + d_{00} >= 2$). The second group, C5-11, deviates some more from the leading eight in the rule $d_{01} = 1$. This rule is also different to C1-4, but all others and the slots of wildcards are the same. However, the additional rule for the wildcard differs. Only one wildcard slot has to be 1 ($c_{00} + d_{10} + d_{00} >= 1$).

The last group, C12-15, deviates from the leading eight in two different rules, $c_{01} = 0$ and $d_{11} = 1$. This means in deviates from C1-4 in the same way and in three ways from C5-11. However, the wildcard slots are the same and the additional rule is also the same as for C1-4: at least two must be good. These specific properties reveal an additional common feature of all C-*. They require at least five rules with positive assessments. (Two of these at are always c_{11} and c_{10}). In comparison, the leading eight have as few as three of such rules. This way the differential equations of C-* are always positive, h-scores grow to 1 and the strategy can reach full cooperation in an homogeneous state. However, they sacrifice some ability to discriminate against defection (which requires negative assessments). Depending on which defections they assess good, these sacrifices allow defectors to invade for different benefit to cost ratios. Concerning other cooperators, no one could ever earn higher payoffs by cooperating more, since C-* have reach full cooperation.

The leading eight established five rules for indirect reciprocity under public assessment. For the 15 ESS strategies under private assessments, we find that the subgroup C1-4, which fit the leading-eight the most (two are identical), are the most successful strategies (i.e. they are stable for the lowest benefit to cost threshold). Only one of these strategies, C-3, disobeys a rule that holds for public assessment, where $d_{10} = 0$ instead of $d_{10} = 1$. For the leading eight, a good player that defects against a bad player should keep his good reputation. This rule was often considered crucial and being referred to as a justified punishment. If no error in execution or assessment occurs, C-3 and others can maintain stable indirect reciprocity without this rule. It is similar for the less successful group C5-11 and the rule $d_{01} = 1$ (instead of $d_{01} = 0$). This assessment rewards defection against good players, which is usually not the case for indirect reciprocity. Similarly, the third group C12-15 rewards the same act (but it is done by good players rather than bad ones, $d_{11} = 1$). Additionally, it is different from leading-eight norms in that it does not reward cooperation by bad players if it is aimed at good players.

References

1. Bortolussi, L., Hillston, J., Latella, D. & Massink, M. Continuous approximation of collective system behaviour: A tutorial. *Performance Evaluation* **70,** 317–349. ISSN: 01665316 (2013).

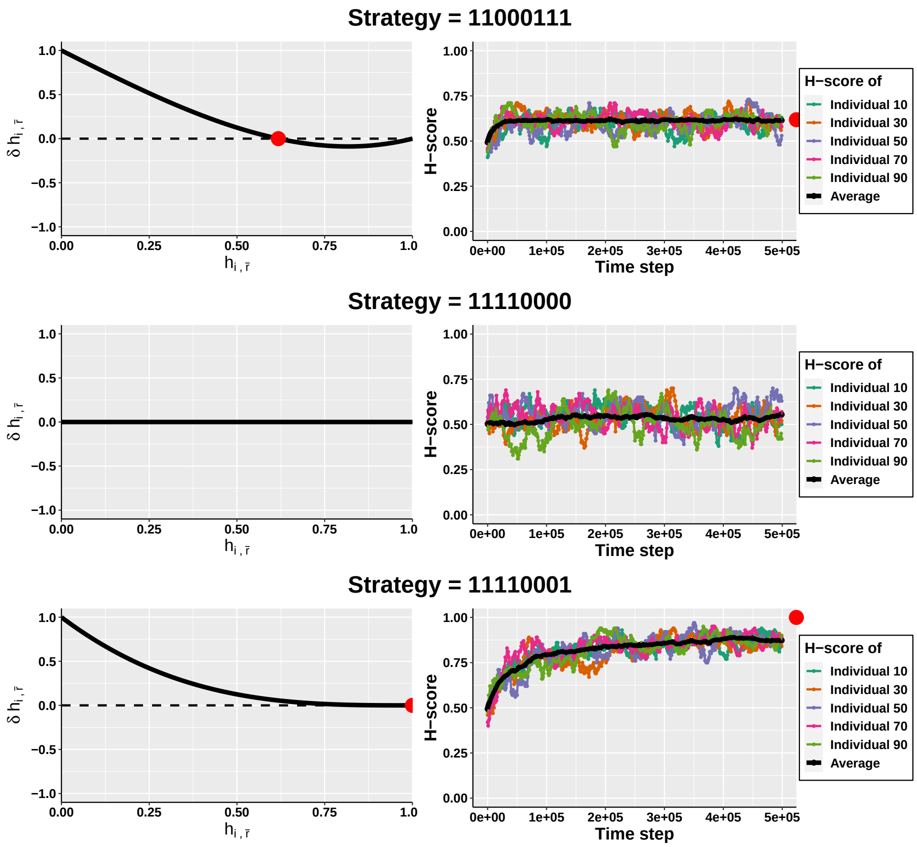

Figure S1: Differential equation (left) and single run of simulations (right) for three different strategies: 10 1100 0111, 10 1111 0000 and 10 1111 0001. The first strategy represents the most common analysis result (around 66%) and the second and third represent particular cases, knowingly when differential equation is equal to 0 (around 3.5% of strategies) and when the equilibrium point is asymptotic (around 30% of strategies). The stable equilibrium points are represented by a red point. They can be found graphically by looking at the points where the function on right hand side of the differential equation intersects with the x-axis. An equilibrium point is stable when the differential equation has a negative slope around the equilibrium point.

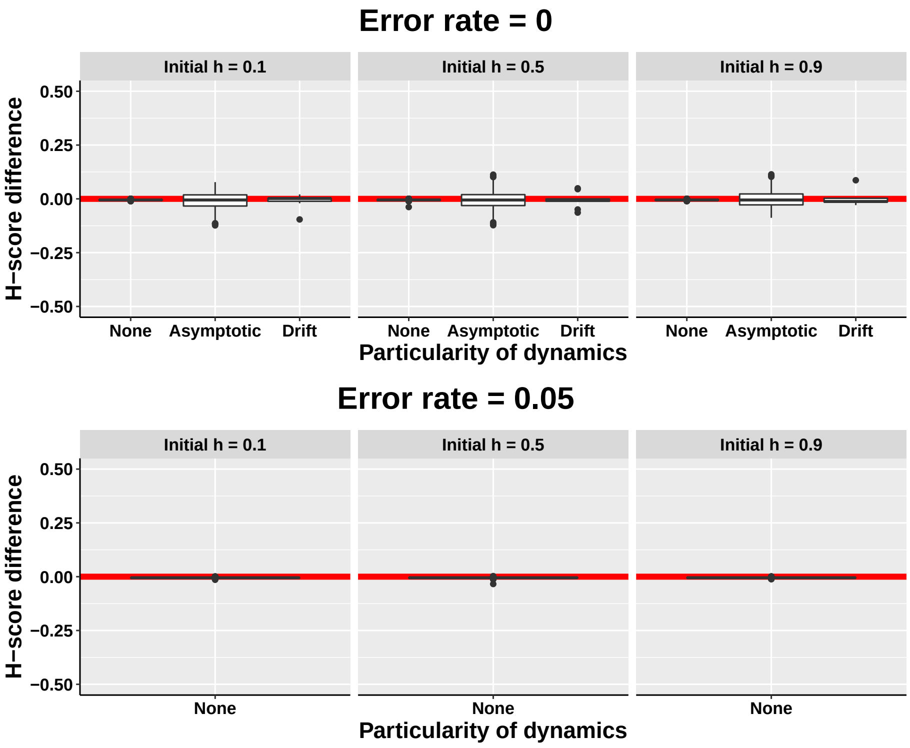

Figure S2: H-score difference between predictions and simulations for different initial h-score. The results are presented for the two cases (i) when the errors are considered negligible in the analytical model, and (ii) when the error rates are significant ($\epsilon = 0.05$). The results are presented as a function of the particularity of the dynamics: "Asymptotic" which represents asymptotic equilibrium points for which the derivative is equal to 0, and "drift" which represents cases where the differential equation is equal to 0. The results presented are the average over 10^5 time step, after 4×10^5 time steps and for 30 replicates. The population size in the simulations is $N = 100$.

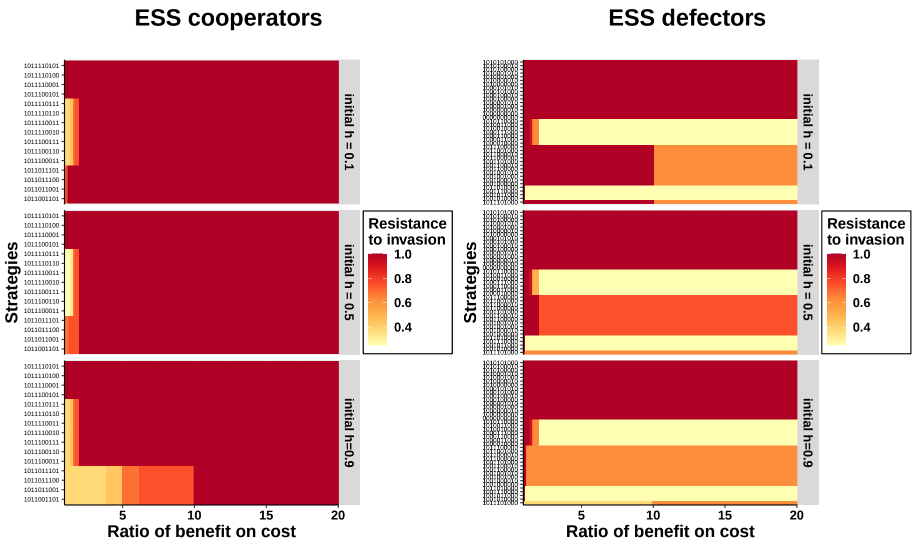

Figure S3: Resistance to invasion as a function of the benefit to cost ratio, b/c, for strategies that are ESS at least once on the parameters' values studied. The left column presents ESS that are cooperators (when ESS) and the right column ESS that are defectors.

2. Otto, S. P. & Day, T. *A biologist's guide to mathematical modeling in ecology and evolution* ISBN: 9780691123448 (Princeton University Press, Princeton, NJ, 2007).

3. Santos, F. C., Santos, F. P. & Pacheco, J. M. Social norm complexity and past reputations in the evolution of cooperation. *Nature* **555,** 242–245. ISSN: 0028-0836 (Mar. 2018).

4. Ohtsuki, H. & Iwasa, Y. How should we define goodness? - Reputation dynamics in indirect reciprocity. *Journal of Theoretical Biology* **231,** 107–120. ISSN: 00225193 (2004).

a_1	a_0	c_{11}	c_{10}	c_{01}	c_{00}	d_{11}	d_{10}	d_{01}	d_{00}	Maximum ratio benefit to cost
1	0	1	0	1	0	1	0	0	0	//
1	0	1	0	1	0	0	0	1	0	//
1	0	1	0	1	0	0	0	0	0	//
1	0	1	0	0	0	1	0	1	0	//
1	0	1	0	0	0	1	0	0	0	//
1	0	1	0	0	0	0	0	1	0	//
1	0	1	0	0	0	0	0	0	0	//
1	0	0	0	1	0	1	0	1	0	//
1	0	0	0	1	0	1	0	0	0	//
1	0	0	0	1	0	0	0	1	0	//
1	0	0	0	1	0	0	0	0	0	//
1	0	0	0	0	0	1	0	1	0	//
1	0	0	0	0	0	1	0	0	0	//
1	0	0	0	0	0	0	0	1	0	//
1	0	0	0	0	0	0	0	0	0	//
0	0	0	0	0	0	0	0	0	0	//
1	0	1	0	1	1	0	0	0	0	1.3
1	0	1	0	0	1	1	0	0	0	1.3
1	0	1	0	0	1	0	0	0	0	1.3
1	0	0	0	1	1	1	0	0	0	1.3
1	0	0	0	1	1	0	0	0	0	1.3
1	0	0	0	0	1	1	0	0	0	1.3
1	0	0	0	0	1	0	0	0	0	1.3
1	0	1	1	1	0	0	0	0	0	1.1
1	0	1	1	0	0	1	0	0	0	1.1
1	0	1	1	0	0	0	0	1	0	1.1
1	0	1	1	0	0	0	0	0	0	1.1
1	0	0	1	1	0	1	0	0	0	1.1
1	0	0	1	1	0	0	0	1	0	1.1
1	0	0	1	1	0	0	0	0	0	1.1
1	0	0	1	0	0	1	0	1	0	1.1
1	0	0	1	0	0	1	0	0	0	1.1
1	0	0	1	0	0	0	0	1	0	1.1
1	0	0	1	0	0	0	0	0	0	1.1
1	0	1	1	0	1	0	0	0	0	1
1	0	0	1	1	1	0	0	0	0	1
1	0	0	1	0	1	1	0	0	0	1
1	0	0	1	0	1	0	0	0	0	1

Figure S4: List of strategies that are defectors and ESS for any initial h-score and a least one value of the benefit to cost ratio, b/c. The last column represents the minimum ratio for which a strategy is ESS for any initial h-score.

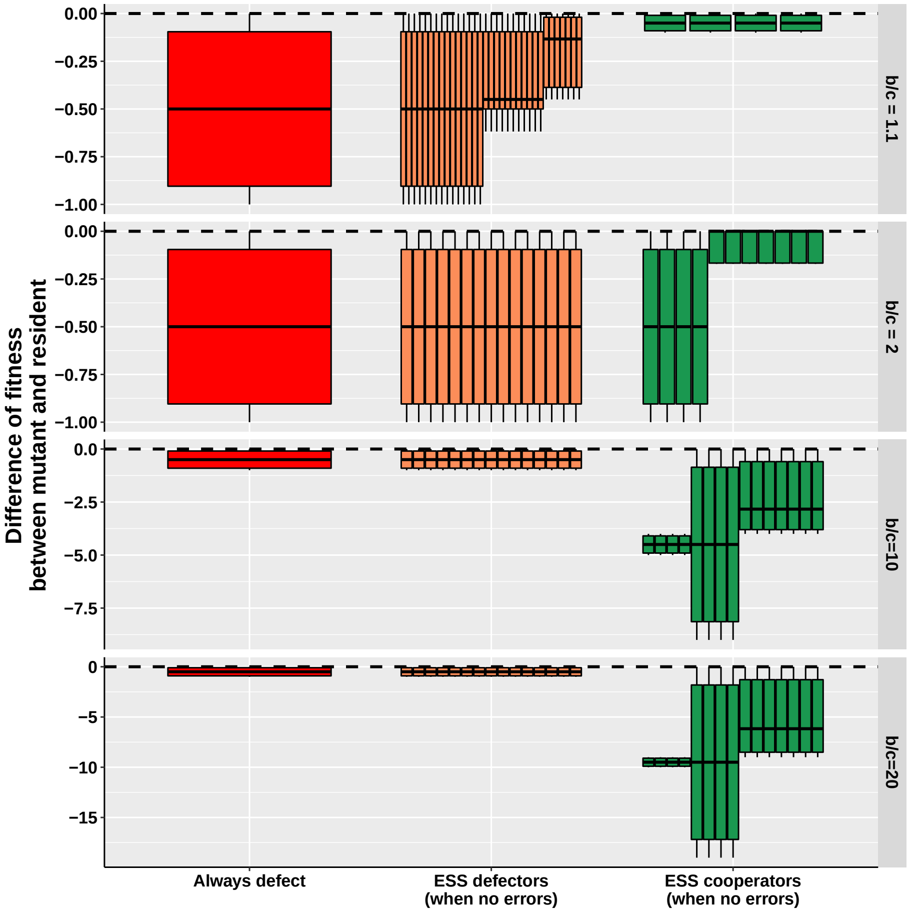

Figure S5: Difference of fitness between mutant and resident $w_m - w_r$, for different strategies that are ESS when there are no errors. We differentiate between strategies that were cooperators, defectors and the strategy that always defect (AllD), which is the only ESS in presence of errors. The results are presented for different benefit to cost ratios b/c.

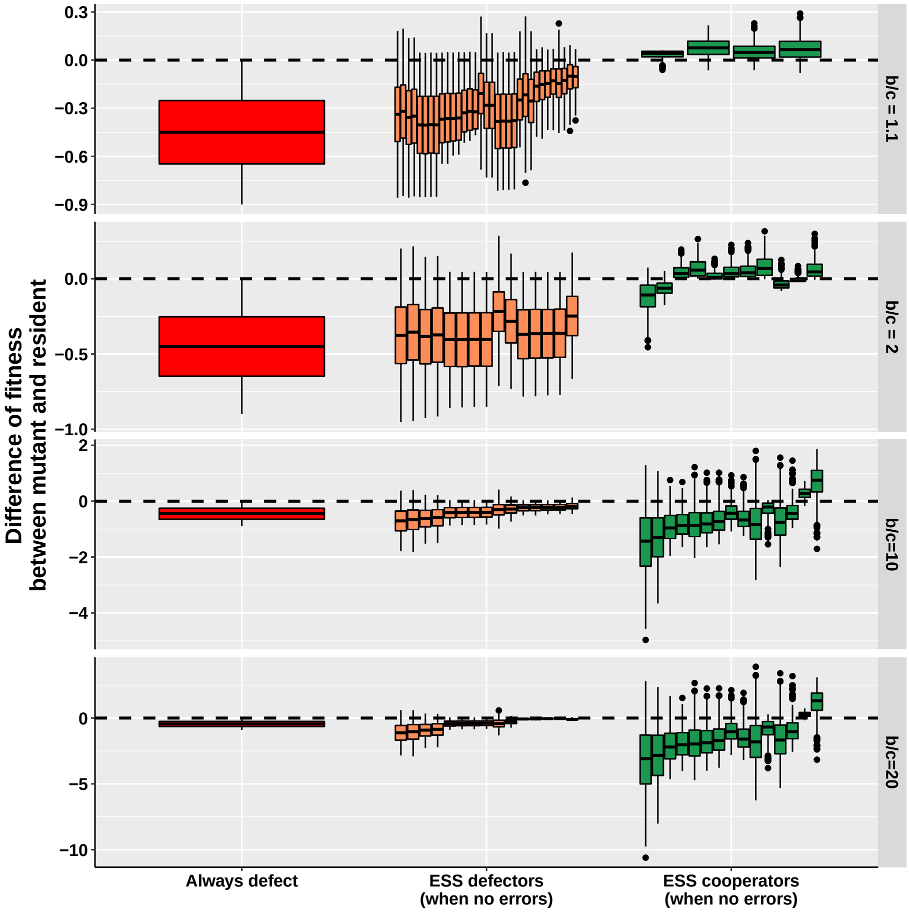

Figure S6: Difference of fitness between mutant and resident $w_m - w_r$, for different strategies that are ESS when there are no errors. We differentiate between strategies that were cooperators, defectors and the strategy that always defect (AllD), which is the only ESS in presence of errors. The results are presented for different benefit to cost ratios b/c.
